# Quantum Router for Single Photons Carrying Spin and Orbital Angular Momentum

**DOI:** 10.1038/srep27033

**Published:** 2016-06-03

**Authors:** Yuanyuan Chen, Dong Jiang, Ling Xie, Lijun Chen

**Affiliations:** 1 State Key Laboratory for Novel Software Technology, Nanjing University, Nanjing, 210046, P.R. China

## Abstract

Quantum router is an essential element in the quantum network. Here, we present a fully quantum router based on interaction free measurement and quantum dots. The signal photonic qubit can be routed to different output ports according to one control electronic qubit. Besides, our scheme is an interferometric method capable of routing single photons carrying either spin angular momentum (SAM) or orbital angular momentum (OAM), or simultaneously carrying SAM and OAM. Then we describe a cascaded multi-level quantum router to construct a one-to-many quantum router. Subsequently we analyze the success probability by using a tunable controlled phase gate. The implementation issues are also discussed to show that this scheme is feasible.

Quantum communication allow people to transmit information quickly over long distance^1^,^2^,^3^,^4^. Compared with classical communication, quantum communication can provide significant improvement of capacities of transmitted information. Therefore, quantum communication has attracted much attention and reaches remarkable achievements in the past decades. In order to implement a multi-user network, the optical switch is used as quantum router^5^,^6^,^7^. Then the wavelength-division multiplexing is used to achieve more flexible network structures^8^,^9^. Besides, Fröhlich *et al.* proposed an upstream network, which can implement multipoint-to-point quantum network with high efficiency^10^. Many quantum routing schemes have also been implemented in experiment^11^,^12^,^13^. However, according to Lemr’s five requirements for quantum router^14^,^15^, most of the existing routers used in quantum networks are just semi-quantum routers. Then Lemr described an all-linear-optical scheme for a fully featured quantum router. Qu *et al.* illustrated an approach for constructing the cascaded multi-level quantum router, which can obtain a *K* level quantum router with 2^*K*^ output ports^16^. But Lemr’s all-linear-optical scheme operates with success probabilities ranging between 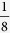 and 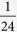 depending on the control qubit state. Consequently the success probability of Qu’s cascaded quantum router would decrease exponentially versus the increase of the number of levels *K*. Overall, an efficient quantum router is an urgent problem that should be solved for the widespread use of quantum networks.

Up to now, most quantum routers are aimed at operating single photons only carrying spin angular momentum (SAM)^17^,^18^,^19^,^20^. In the past decades, orbital angular momentum (OAM) has attracted much attention as the Hilbert space spanned by these states is in principle infinite. Thus it can tremendously increase the capacity of communication system. A large number of researches based on OAM, such as the generation and manipulation^21^, quantum teleportation^22^ and optical communications^23^,^24^, have been conducted. Thus single photons carrying both SAM and OAM could be a more efficient information carrier in the future quantum network. Consequently, an efficient quantum router, which can route single photons carrying either SAM or OAM, or simultaneously carrying SAM and OAM, is requisite.

In this work, by using interaction free measurement^25^ and quantum dots^26^,^27^, we present a fully quantum router for single photons with high efficiency. We first describe the structure of the proposed quantum router, which can route single photons according to the state of the control quantum qubit. Here we use Mach-Zehnder interferometer^28^ to implement interaction free measurement. The quantum dot (QD) is used for changing the phase of input signal photon, which could lead the superpositions in two paths of Mach-Zehnder interferometer to produce phase difference. Due to the principle of interaction free measurement, the phase difference in two superpositions would make the input signal photon pass through different output ports. Then in order to get a one-to-many quantum router, we use a cascaded method to obtain a *K* level quantum router with 2^*K*^ output ports. Subsequently, the success probability of the proposed quantum router is analyzed by means of a tunable control phase gate. Finally, we discuss the implementation issues to show that our scheme can be realized in experiment.

## Results

In this section, we use interaction-free measurement and quantum dot to construct an efficient quantum router. The interaction-free measurement is realized by a Mach-Zehnder interferometer, where a quantum dot is placed on one path. Here the singly charged GaAs/InAs quantum dot^27^ is used to change the photon’s trajectory. That is to say the transmission direction of signal input is controlled by a quantum method rather than a classical method. Moreover, this quantum router can be extended to multiple output ports by constructing cascaded quantum router.

### Quantum router for single photons carrying spin angular momentum

**Figure 1:**
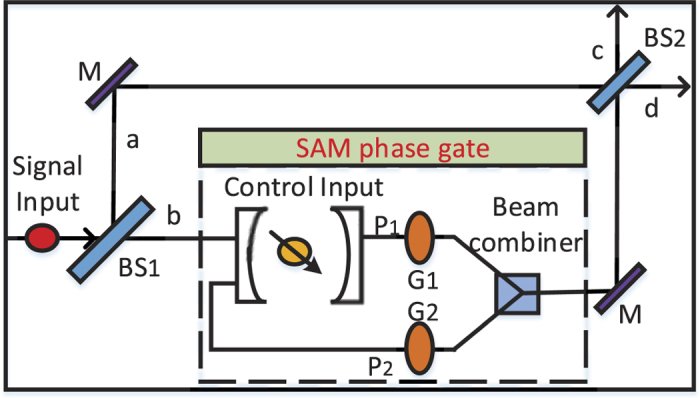
A schematic illustration of quantum router for single photons carrying spin angular momentum. BS: beam splitter, M: mirror, *G*_1,2_: phase shifter. The beam combiner is used to allow the superpositions in two paths to simultaneously pass through *BS*_2_. SAM phase gate can shift phase according to the state of control input quantum qubit.

As shown in Fig. 1, the input signal takes the form: |*ψ*_*s*_〉 = *α*|*H*〉 + *β*|*V*〉, where |*H*〉 and |*V*〉 denote the states of horizontal and vertical linear polarization, |*α*|^2^ + |*β*|^2^ = 1. Here we may use SAM to instead of the term “polarization” for simplicity. Then the photon is sent towards the beam splitter, whose reflectivity and transmission are 50:50. So the state of the system becomes:


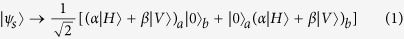


where |0〉_*a*/*b*_ represents that no photon is transmitted through the path *a*/*b*. Here we consider a double sided cavity system, in which a singly charged QD is embedded. Initially, an absorption electron in state |*ϕ*_*c*_〉 = *γ*|↑〉 + *δ*|↓〉 was placed in the coupled double quantum dot system. According to the optical selection rules^29^ and the transmission and reflection rules of the cavity^27^ (see Methods for detail), the left circularly polarization photon (|*L*〉) only couples the electron in the spin state |↑〉. While the right circularly polarization photon (|*R*〉) only couples the electron in the spin state |↓〉. Here, the quarter-wave plate can achieve the photon’s transformation between linear and circular polarization |*L*〉 (|*R*〉) ↔ |*H*〉 (|*V*〉). Therefore, for an incident photon in state |*H*〉, if the electron is |↑〉, the photon will be reflected by the cavity. On the other hand, if the electron spin is |↓〉, the photon is transmitted through the cavity. Similarly, for an incident photon in state |*V*〉, if the electron spin is |↑〉, the photon is transmitted through the cavity. Otherwise, if the electron spin is |↓〉, the photon will be reflected by the cavity. Based on the rules discussed above, the state *α*|*H*〉 + *β*|*V*〉 will interact with the QD as:


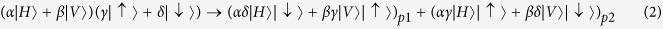


Then the two quantum gates in path *P*_1_ and *P*_2_ will change the state as: *G*_1_: |*H*〉 → |*H*〉, |*V*〉 → −|*V*〉 and *G*_2_: |*H*〉 → −|*H*〉, |*V*〉 → |*V*〉. Therefore, the system state before *BS*_2_ is:


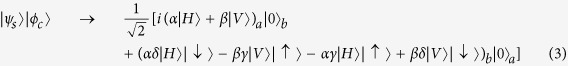


The reflectivity and transmission of *BS*_2_ are also 50:50. The system state after *BS*_2_ can be expressed as:


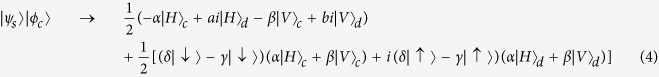


As usual, the control electron impinges on the detector. Depending on the outcome of the detection measurement performed, the signal qubit collapses into:


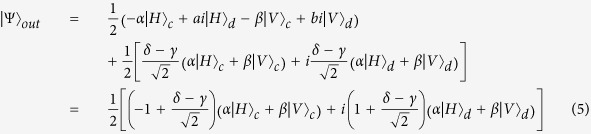


The signal information is unchanged under the routing operation, while the spatial degree of freedom is modified depending on the parameter *γ* and *δ* of the control qubit. As shown in Eq. (5), if 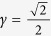, 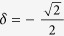, the signal photon will pass through output port *c*. If 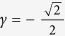, 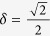, the signal photon will pass through output port *d*. If 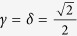, the signal photon will be in spatial superposition of port *c* and port *d*.

### Quantum router for single photons carrying orbital angular momentum

**Figure 2:**
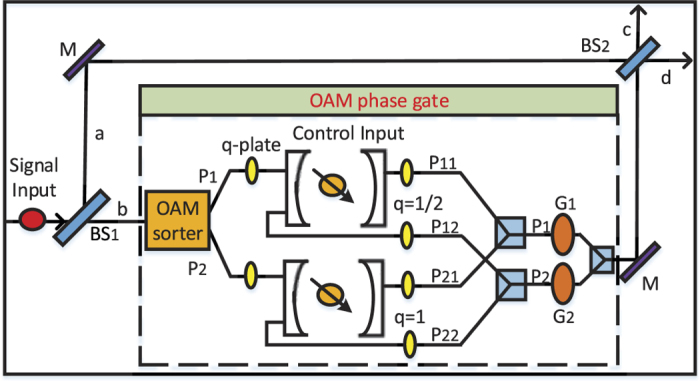
Experimental setup of quantum router for single photons carrying orbital angular momentum. OAM sorter can direct photon superposition according to the OAM value without disturb the quantum state. The q-plate device can be used as a coherent and bidirectional quantum interface allowing the transfer of quantum information between the polarization and the orbital angular momentum degrees of freedom of the photon. BS: beam splitter, M: mirror, *G*_1,2_: phase shifter. OAM phase gate can shift phase according to the state of control input quantum qubit.

Different from polarization, orbital angular momentum contains multiple degrees of freedom. Here we propose a interferometric scheme to realize a quantum router for single photons with orbital angular momentum. As shown in Fig. 2, the input signal takes the form: |Ψ_*o*_〉 = *α*_1_|1〉 + *β*_1_|−1〉 + *α*_2_|2〉 + *β*_2_|−2〉, where |*α*_1_|^2^ + |*β*_1_|^2^ + |*α*_2_|^2^ + |*β*_2_|^2^ = 1. Then the photon is sent towards the beam splitter, whose reflectivity and transmission are 50:50. So the state becomes:


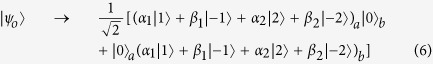


where |0〉_*a*/*b*_ represents that no photon is transmitted through the path. Here the photon in path *b* will pass through an orbital angular sorter proposed by Leach, which is used to measuring the value of orbital angular momentum 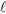 without disturbing its superposition state. After orbital angular momentum sorter, the photon in path *b* will become (*α*_1_|1〉 + *β*_1_|−1〉)_*p*1_ + (*α*_2_|2〉 + *β*_2_|−2〉)_*p*2_. Then the q-plates are used to realize the transformation between circular polarization and orbital angular momentum as


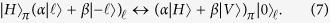


The q-plates in two paths would change the photon state in path *b* into 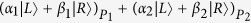 (The quarter-wave plate can be used to perform the transformation |*H*〉 (|*V*〉) ↔ |*L*〉 (|*R*〉)). Next, we still use the singly charged QD embedded in double sided cavity system to control the router. For simplicity, the two quantum dots in two paths are prepared in the same superposition state |*ϕ*_*c*_〉 = *γ*|↑〉 + *δ*|↓〉. According to the Pauli spin blockade, the states *α*_1_|*L*〉 + *β*_1_|*R*〉 and *α*_2_|*L*〉 + *β*_2_|*R*〉 will interact with the QD as:


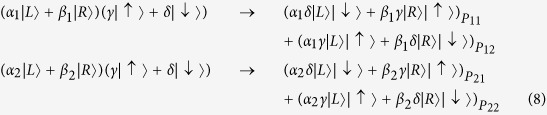


Then both the superposition states in two paths pass through the corresponding q-plate to transfer circular polarization to orbital angular momentum. So the state will become


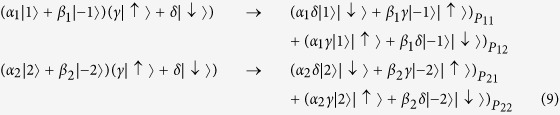


After that, paths *P*_11_ and *P*_21_ are led to *P*_1_, while paths *P*_12_ and *P*_22_ are led to *P*_2_. So the two quantum states in path *P*_1_ and *P*_2_ will become


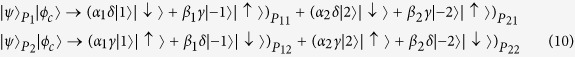


Here we need to change the state as: *G*_1_: |*k*〉 → |*k*〉, |−*k*〉 → −|−*k*〉 and *G*_2_: |*k*〉 → −|*k*〉, |−*k*〉 → |−*k*〉, where *k* = 1 or 2. Therefore, the state before *BS*_2_ is:


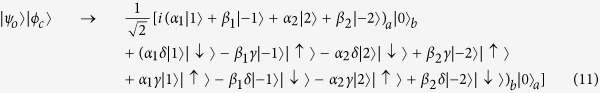


The reflectivity and transmission of *BS*_2_ are also 50:50. The system state after *BS*_2_ can be expressed as:


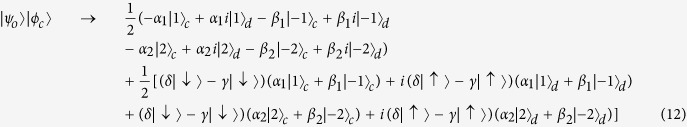


As usual, the control electrons impinge on the detector. Depending on the outcome of the detection measurement performed, the signal qubit collapses into:


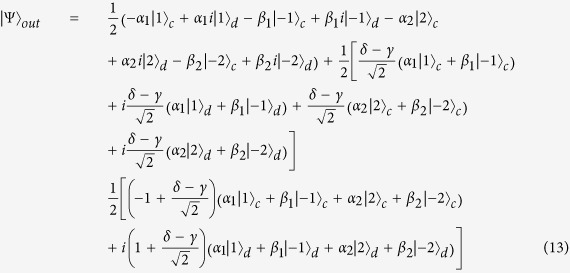


Similarly, the signal information is unchanged under the routing operation, while the spatial degree of freedom is modified depending on the parameter *γ* and *δ* of the control electron qubit.

### Quantum router for photons carrying spin and orbital angular momentum

**Figure 3:**
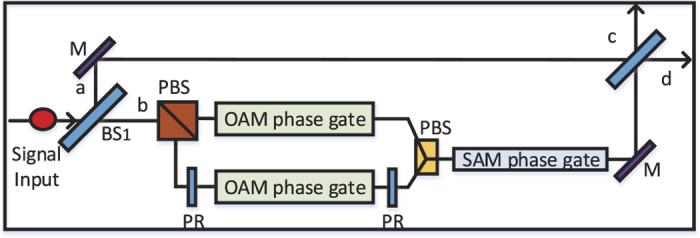
The model of quantum router for single photons carrying spin and orbital angular momentum. BS: beam splitter, M: mirror, PR: polarization rotator, PBS: polarization beam splitter. OAM phase gate can shift phase according to the state of control input quantum qubit without disturbing the SAM value. While SAM phase gate can shift phase according to the state of control input quantum qubit without disturbing the OAM value.

When the incident photon carries both spin and orbital angular momentum, we also can use an interferometric method to construct a quantum router. As shown in Fig. 3, the input signal takes the form: 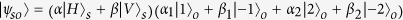, where |*α*|^2^ + |*β*|^2^ = 1, |*α*_1_|^2^ + |*β*_1_|^2^ + |*α*_2_|^2^ + |*β*_2_|^2^ = 1. Then the photon in path *b* is sent towards the polarization beam splitter, which transmits horizontal polarization and reflects vertical polarization. As discussed above, the input photon of OAM phase gate is required to be horizontal polarization. So the polarization rotator is used to transform vertical polarization into horizontal polarization. After the photon passing through OAM phase gate, the polarization rotator transforms horizontal polarization back to vertical polarization. Thus the state of the system will become:


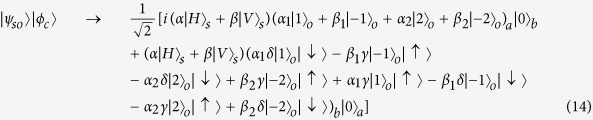


Then the photon passes through SAM phase gate, the state will become:


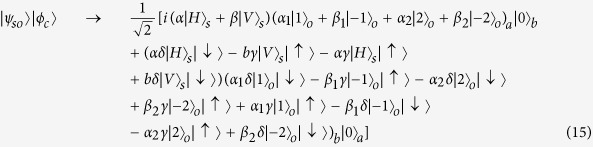


The reflectivity and transmission of *BS*_2_ are also 50:50, thus the system state after *BS*_2_ can be expressed as:


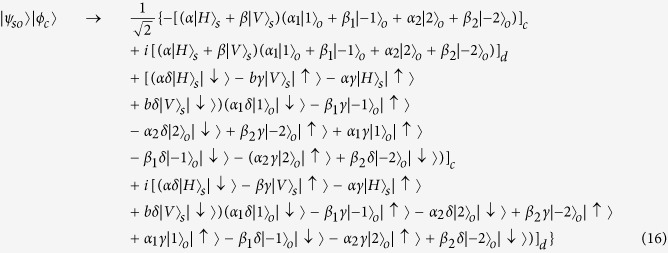


As usual, the control electrons impinge on the detector. Depending on the outcome of the detection measurement performed, the signal qubit collapses into:


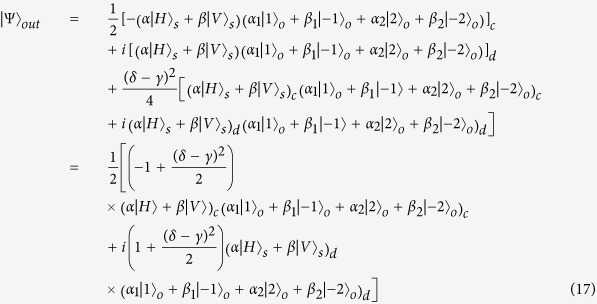


Similarly, the signal information is unchanged under the routing operation, while the spatial degree of freedom is modified depending on the parameter *γ* and *δ* of the control qubit.

### Cascaded multi-level quantum router

**Figure 4:**
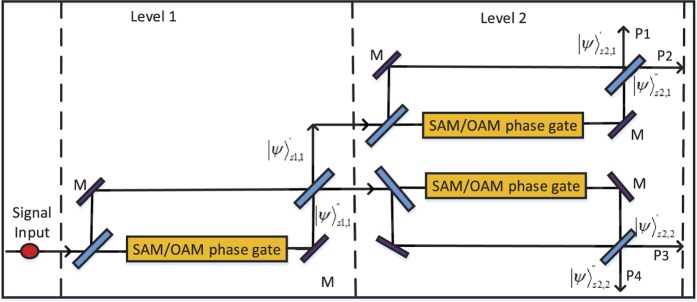
The model of cascaded multi-level quantum router. A 2-level quantum router with 4 output ports is showed.

Here we describe an approach for constructing the cascaded multi-level quantum router. Inspired by Qu’s work^16^, the signals in the router outports of the *i*th level can be regarded as the input signals of the (*i* + 1)th level. As shown in Fig. 4, the two output ports of the 1st level are connected to the input ports of the 2nd level. According to the discussion above, we know the input signals of the 2nd level can be expressed as:


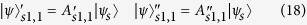


Therefore, the states of each port in 2nd level can be written as:


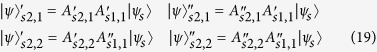


The whole state of the system after two-level quantum router can be described as:


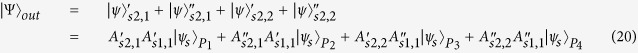


For photons carrying spin angular momentum, 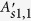 and 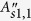 can be obtained from Eq. (5):


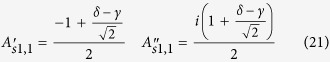


Then the output state after a 2-level quantum router is:


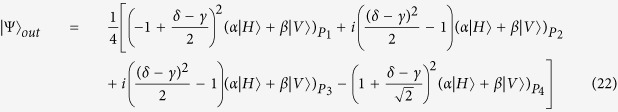


Similarly, for photons carrying orbital angular momentum, 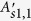 and 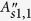 can be obtained from Eq. (13):


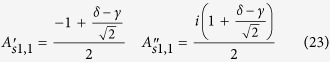


Then the output state after a 2-level quantum router is:


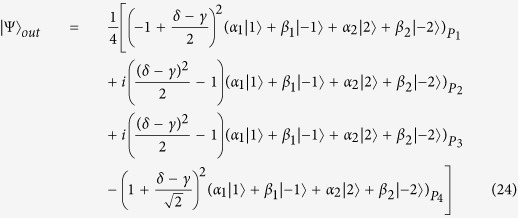


While for photons carrying spin and orbital angular momentum, 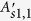 and 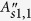 can be obtained from Eq. (17):


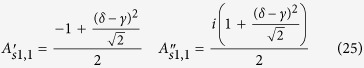


Then the output state after a 2-level quantum router is:


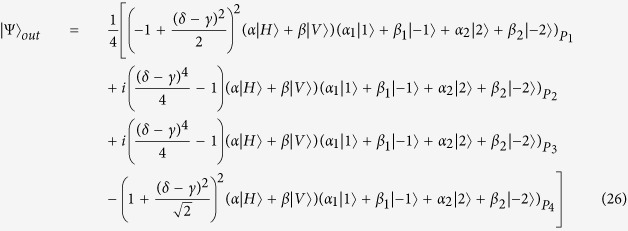


Obviously, the cascaded multi-level quantum router is successfully implemented. In this way, we can obtain a *K* level quantum router with 2^*K*^ output ports.

## Discussion

We discuss the performance of the proposed quantum router by numerically analyzing the success probability. The success probability of quantum router for photons carrying SAM is equal to that of the quantum router for photons carrying OAM. As discussed above, the output port of the 1st level quantum router is expressed as Eq. (5) and Eq. (13). So the success probability of one-level quantum router for photons carrying either SAM or OAM is:


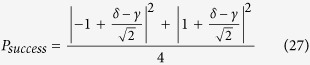


where |*γ*|^2^ + |*δ*|^2^ = 1. When |*γ*| = |*δ*|, the photon is led to two output ports with equal probability. In this case, the success probability gets the minimal value 1/2. While 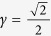, 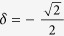 or 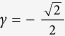, 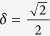, the success probability gets the maximal value 1. For cascaded multi-level quantum router, we assume that the control qubit are both in the state |*ϕ*_*c*_〉 = *γ*|↑〉 + *δ*|↓〉, where |*γ*|^2^ + |*δ*|^2^ = 1. Thus, the success probability of two-level quantum router for photons carrying either SAM or OAM is:


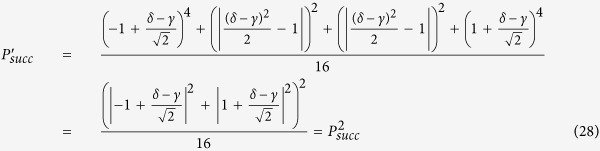


**Figure 5:**
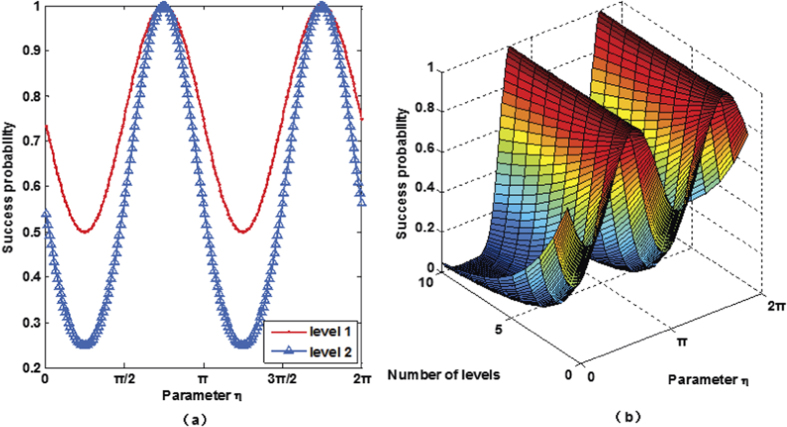
(**a**) Success probability of quantum router for single photons carrying either SAM or OAM as a function of the parameter *η*. The success probability of one-level quantum router ranges between 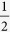 and 1. While the success probability of two-level quantum router ranges between 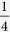 and 1. (**b**) Success probability of cascaded quantum router for single photons carrying either SAM or OAM as a function of the parameter *η* and the number of levels.

So the proposed two-level quantum router operates with success probabilities ranging between 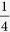 and 1 depending on the control qubit. The plot in Fig. 5(a) shows the success probability of routing as a function of parameter *η* (*γ* = cos *η*, *δ* = sin *η*).

Next we consider the success probability of multi-level quantum router for photons carrying either SAM or OAM. Suppose that the two output states in the *i*th level and the *j*th quantum router are 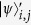 and 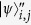. Then we can get the four corresponding output states in the (*i* + 1)th level:


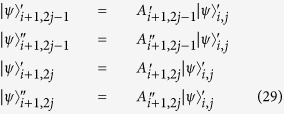


If the control qubits of all the routers are the same, we can get the success probability of the *m*-level quantum router 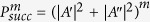. The success probability of the cascaded quantum router versus parameter *η* is described in Fig. 5(b).

Different from the above discussion, the output port of the 1st level quantum router is expressed as Eq. (17). So the success probability of one-level quantum router for photons carrying both SAM and OAM is:


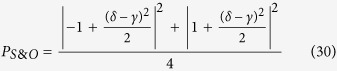


When |*γ*| = |*δ* |, the photon is led to two output ports with equal probability. In this case, the success probability gets the minimal value 1/2. While 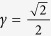, 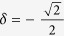 or 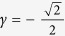, 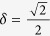, the success probability gets the maximal value 1. Thus, the success probability of two-level quantum router for photons carrying both SAM and OAM is:


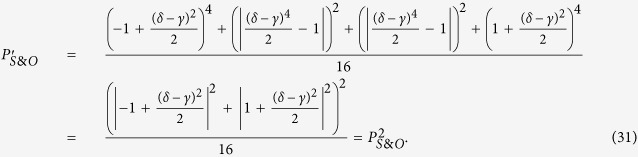


**Figure 6:**
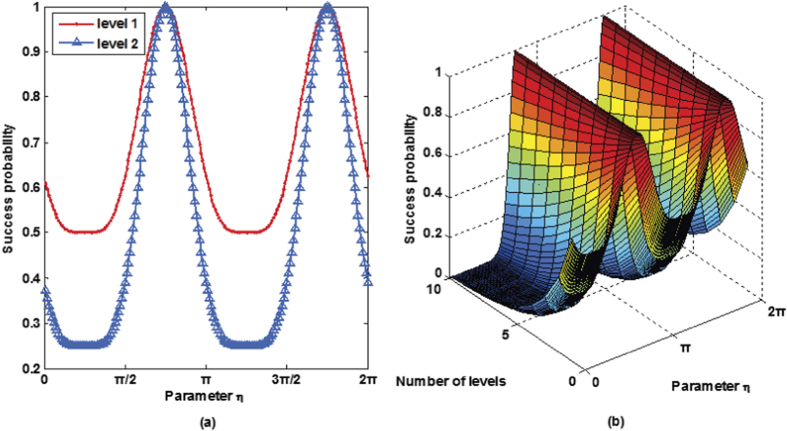
(**a**) Success probability of quantum router for single photons carrying both SAM and OAM as a function of the parameter *η*. The success probability of one-level quantum router ranges between 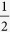 and 1. While the success probability of two-level quantum router ranges between 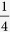 and 1. (**b**) Success probability of cascaded quantum router for single photons carrying both SAM and OAM as a function of the parameter *η* and the number of levels.

So the proposed two-level quantum router for photons carrying both SAM and OAM operates with success probabilities ranging between 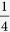 and 1 depending on the control qubit. The plot in Fig. 6(a) shows the success probability of routing as a function of parameter *η*.

Similarly, when the present scheme is extended to multi-level, the success probability will be 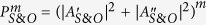, where *m* is the number of levels. Figure 6(b) describes the success probability of the cascaded quantum router versus parameter *η*.

**Figure 7:**
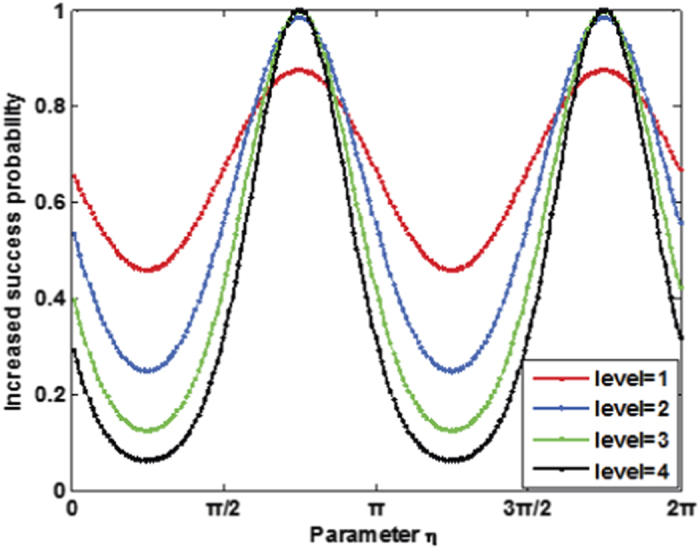
The increased success probability by comparing the success probability of our scheme and Lemr’s scheme.

Compared with Lemr’s scheme^14^, our quantum router is more efficient. As shown in Fig. 7, the increased success probability depends on the control electronic qubit. Due to the high efficiency, our proposed quantum router makes commercial applications conceivable.

## Methods

In order to implement the proposed quantum router, we need to realize two main operations: SAM and OAM phase gate and OAM sorter. In our scheme, SAM and OAM phase gates are controlled and manipulated by a quantum dot. So we use the singly charged GaAs/InAs quantum dot to serve as a tunable phase gate.

**Figure 8:**
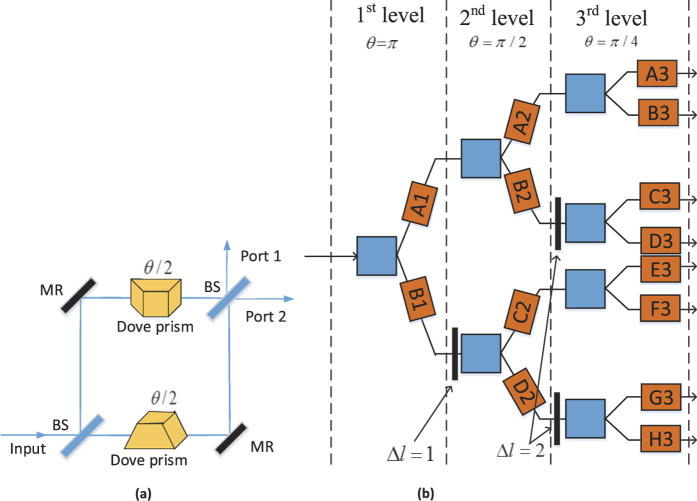
Experimental setup of OAM sorter. BS: beam splitter, MR: mirror and 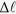 represents that 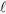 is added to 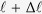. (**a**) The input photon will be splitted into two arms by BS. The two dove prism in two arms result in a phase rotation of *θ*. If the phase difference Δ*ω* = 2*kπ*, the photon will pass through port 1. On the other hand, if Δ*ω* = (2*k* + 1)*π*, *k* is integer, the photon will pass through port 2. (**b**) In level 1, *θ* is set to be *π*, the photon with even 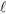 will pass through port A2, while the photon with odd 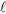 will pass through port B2. Similarly, all values of 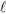 can be classified into different ports.

Here we use Leach’s interferometric method to realize OAM sorter^30^. As shown in Fig. 8(a), the superposition states in the two arms are rotated with respect to each other through an angle *θ*/2. The initial transfer mode of the photon is 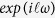. After being rotated through an angle *θ* by the Dove prism, the phase becomes 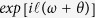, which results in a phase shifter of 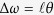. According to the principle of Mach-Zehnder interferometer, if Δ*ω* = 2*kπ*, *k* = 1, 2 …, the photon would pass through port 1. Otherwise, if Δ*ω* = (2*k* − 1)*π*, *k* = 1, 2 …, the photon would pass through port 2. Therefore, we can construct a device, as shown in Fig. 8(b), to classify the OAM values. In Level 1, as *θ* = *π*, if the OAM value is even, the photon will pass port *A*_2_. If the OAM value is odd, the photon will pass port *B*_2_. The results of other levels are described in Table 1 (k = 1, 2, 3 …).

**Figure 9:**
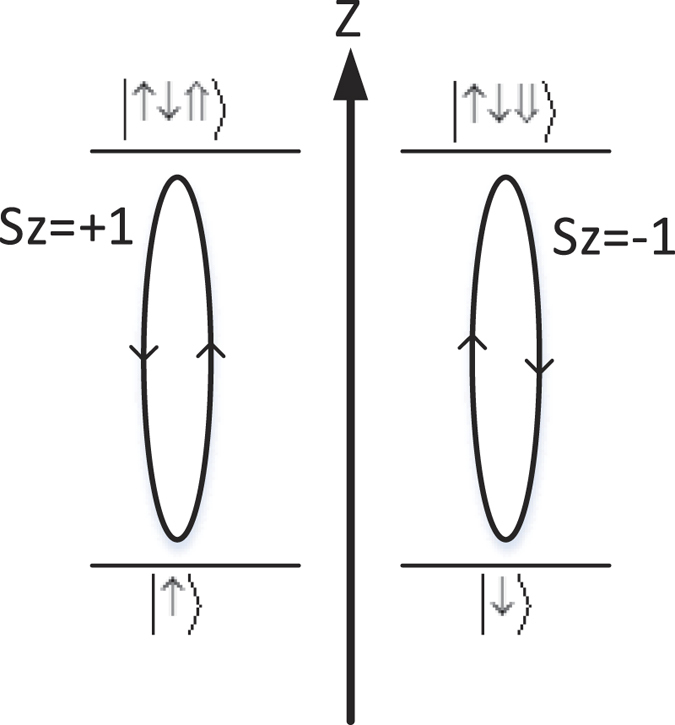
Relevant energy levels and optical selection rules for the optical transition of negatively charged exciton *X*^−^.

Recently, Hu *et al.*^27^ proposed a singly charge self-assembled GaAs/InAs quantum dot being embedded in an optical resonant double-sided microcavity, which has been recognized by Bonate *et al.*^31^. The singly charged quantum dot has four relevant electronic levels, |↑〉, |↓〉, 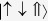 and 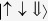 as shown in Fig. 9. Here the symbols 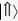 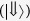 and |↑〉 (|↓〉) represent a heavy hole and an electron with *Z*-direction spin projections 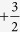 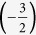 and 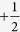 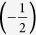, respectively. The optical excitation of the system can produce an excitation with negative charges and the charged exciton *X*^−^, which consists of two electrons bound in one hole. According to the optical selection rules and the transmission and reflection rules of the cavity for an incident circular polarization with *S*_*z*_ = ±1 conditioned on the QD-spin state, the interaction between photon and electrons in the QD-microcavity coupled system is described as below:


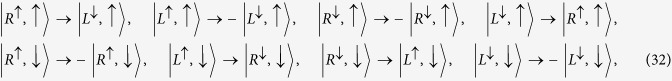


where the superscript arrow in the photon state indicates the propagation direction along the *Z* axis. Therefore, for the incident photon with spin |−1〉 (|*R*^↓^) *or* |*L*^↑^〉), if the electron is in the state |↑〉, there is no dipole interaction and the photon is transmitted through the cavity. On the other hand, if the electron is in the state |↓〉, the photon will couple with the electron and be reflected by the cavity. Then the photon state is transformed into the state |*L*^↑^〉 or |*R*^↓^〉, respectively. Similarly, a photon with spin | + 1〉 (|*R*^↑^〉 *or* |*L*^↓^〉) will be transmitted when the electron-spin state is |↓〉 and will be reflected by the cavity when the electron-spin state is |↑〉. Therefore, the quantum-dot-microcavity system is very suitable to serve as the quantum qubit to control and manipulate the SAM and OAM phase gate.

## Additional Information

**How to cite this article**: Chen, Y. *et al.* Quantum Router for Single Photons Carrying Spin and Orbital Angular Momentum. *Sci. Rep.* **6**, 27033; doi: 10.1038/srep27033 (2016).

## Figures and Tables

**Table 1:** Results of OAM sorter.

Level 2	port *A*_2_	port *B*_2_						
	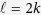	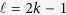						
Level 3	port *A*_3_	port *B*_3_	port *C*_3_	port *D*_3_				
	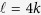	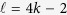	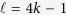	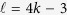				
Level 4	port *A*_4_	port *B*_4_	port *C*_4_	port *D*_4_	port *E*_4_	port *F*_4_	port *G*_4_	port *H*_4_
	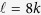	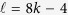	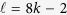	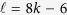	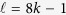	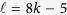	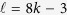	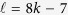
